# Neuroprotective properties of exosomes and chitosan nanoparticles of Tomafran, a bioengineered tomato enriched in crocins

**DOI:** 10.1007/s13659-023-00425-9

**Published:** 2024-01-12

**Authors:** Mikel Etxebeste-Mitxeltorena, Enrique Niza, Cristián Martinez Fajardo, Carmen Gil, Lourdes Gómez-Gómez, Ana Martinez, Oussama Ahrazem

**Affiliations:** 1 Centro de Investigaciones Biológicas Margarita Salas, Consejo Superior de Investigaciones Científicas, C/Ramiro de Maeztu, 9, 28040 Madrid, Spain; 2 Instituto Botánico, Universidad de Castilla-La Mancha, Campus Universitario s/n, 02071 Albacete, Spain; 3 Facultad de Farmacia, Departamento de Ciencia y Tecnología Agroforestal y Genética, Universidad de Castilla-La Mancha, Campus Universitario s/n, 02071 Albacete, Spain; 4 Centro de Investigación Biomédica en Red de Enfermedades Neurodegenerativas (CIBERNED), Instituto de Salud Carlos III, 28031 Madrid, Spain; 5 Escuela Técnica Superior de Ingeniería Agronómica y de Montes y Biotecnología. Departamento de Ciencia y Tecnología Agroforestal y Genética, Universidad de Castilla-La Mancha, Albacete, Spain

**Keywords:** Alzheimer’s disease, Saffron, Tomato, Crocins, Tomafran, Neuroprotection

## Abstract

Saffron has many pharmacological properties in addition to being a frequently used food seasoning. Crocin and picrocrocin which accumulate in saffron stigma, are responsible for these pharmacological properties. These natural products have health‐promoting effects for the prevention and treatment of numerous diseases, including age‐related cognitive and memory disfunction. Currently, crocin and picrocrocin are obtained from saffron, considered as the spice with the highest price in the market. To develop an efficient and low‐cost approach to producing these compounds with high bioactivity, biosynthetic genes isolated from saffron can be exploited in the metabolic engineering of heterologous hosts and the production of crocins in productive crop plants. Recently, we engineered tomato fruit producing crocins (Tomafran). In this study, we demonstrated that crocin-rich extract, encapsulated in chitosan or in exosomes may function as a neuroprotective strategy. Crocins contained in the Tomafran extracts and much lower doses in chitosan nanoparticles or exosomes were enough to rescue the neuroblastoma cell line SH-SY5Y after damage caused by okadaic acid. Our results confirm the neuroprotective effect of Tomafran and its exosomes that may be useful for the delay or prevention of neurodegenerative disorders such as Alzheimer’s disease.

**Graphical Abstract:**

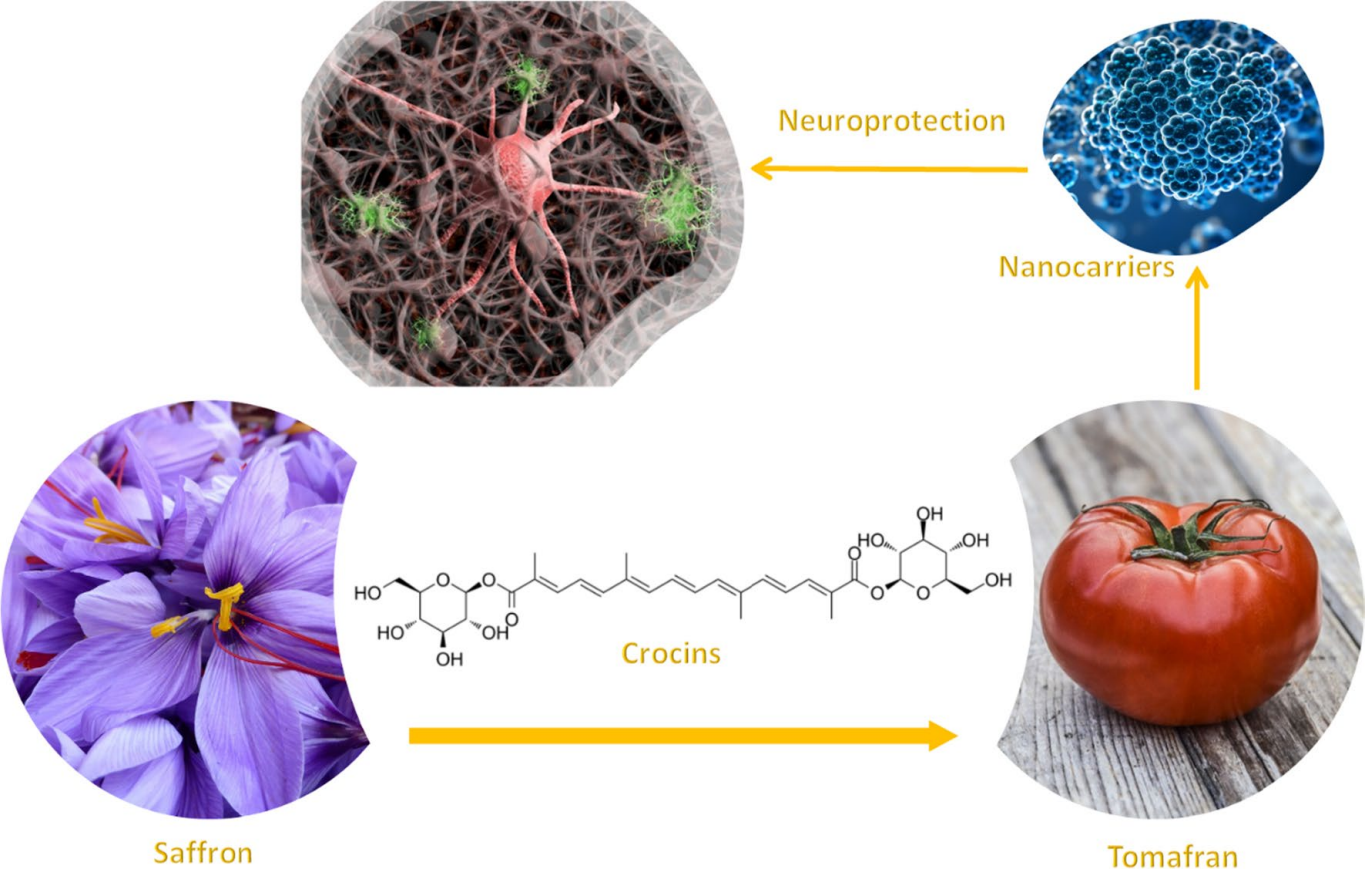

## Introduction

Neurodegenerative diseases are a group of disorders characterized by the progressive degeneration and loss of function of neurons in the brain and, in some cases, the spinal cord [1]. These diseases primarily affect the structure and function of the nervous system, leading to a decline in cognitive function, movement, and overall neurological health. Some common neurodegenerative diseases include Alzheimer's disease (AD) the most common form of dementia that is characterized by the accumulation of abnormal protein clumps (amyloid plaques and tau tangles) in the brain. Main clinical symptoms include memory loss, cognitive decline, and behavioral changes [2].

The exact causes of neurodegenerative diseases are often complex and multifactorial, involving a combination of genetic and environmental and lifestyle factors, being ageing the main risk factor [3]. Diagnosis is typically based on clinical symptoms, neurological examinations, and sometimes imaging or laboratory tests. Treatment options for neurodegenerative diseases are limited, and most of them are focused on managing symptoms and improving patients quality of life [4]. Nowadays, few drugs are approved for these devastating diseases, therefore, discovering an effective treatment for these pathologies are highly needed.

Herbal medicine can be a novel and supplementary kind of treatment. About 80% of people utilize herbal treatments for primary healthcare, mostly in underdeveloped nations. *Ginkgo biloba*, *Huperzia serrata*, *Salvia officinalis*, *Melissa officinalis*, and *Crocus sativus *L. (saffron) are a few significant herbal plants extensively used for brain health and memory [5].

Carotenoids, a group of pigments found in several fruits, vegetables, and other organisms, play an important role in human nutrition. They have antioxidant properties and are associated with health benefits, such as healthy vision, support of the immune system, and risk reducing of certain diseases [6].

Apocarotenoids, compounds derived from carotenoids through enzymatic or chemical modifications, have also important biological functions such as signaling molecules, pigments, and defense compounds, being interesting targets for research in fields such as plant biology, natural product chemistry, and human health [7].

Among these apocarotenoids, crocetin and its glycosidic derivatives crocins, responsible for the red color of saffron, have gained attention for their potential health benefits. Indeed, different research suggested that both crocetin and crocins may have neuroprotective effects, being also investigated for their potential anti-cancer properties, cardiovascular benefits and anti-diabetic potential [8].

Due to the biological interest of these compounds, different strategies have been recently used to modify tomato fruits, tobacco plants, and potato tubers to express specific saffron genes, which allow the biosynthesis and accumulation of high amounts of crocin and picrocrocin [9–11].

Nowadays, saffron is mainly used by the pharma industry for its antioxidant properties and can be found in various food supplements. However, new uses have started to be exploited such as its neuroprotectant activity in AD preclinical models [12, 13]. Growing demand for the product from the pharmaceutical sector is a major trend influencing saffron market dynamics. To respond to this growing demand, Tomafran, an engineered tomato that exclusively accumulates crocins and picrocrocin in the fruit was generated in our group [11], thus opening up the possibility to use tomafran extracts containing crocins in different pharma industry at low costs.

One important challenge in neuroprotective strategies is the cross of blood–brain barrier (BBB) necessary to reach the central nervous system (CNS) and produce the desired biological effect. In this sense, nanotechnology and nano-devices arises as new approach to overcome the problematic uptake of several drugs through BBB and achieve a targeted delivery of many drugs improve the treatment efficacy [14]. Chitosan, an FDA-approved natural cationic polysaccharide obtained from natural sources widely used to obtain biodegradable and biocompatible nanoparticles, can enhance drug permeability across the BBB [15]. Another different strategy involves the use of plant-derived exosomes which are small membrane-bound vesicles released by plant cells. They contain a variety of biological molecules, including proteins, lipids, nucleic acids and small metabolites with important roles in intercellular communication within plants and other organisms [16]. Furthermore, plant exosomes have attracted attention for their potential applications in agriculture, such as crop improvement, disease resistance, and nutrient transfer and medicine such as a novel drug-delivery system [17].

In an attempt to prove the neuroprotective effects of Tomafran and the blood–brain barrier permeability, in this work we used extracts from saffron, tomato and Tomafran, encapsulated Tomafran crocins into biocompatible nanoparticles and exosomes purified from Tomafran. These samples were tested in a well-known cellular model of AD based in human neuroblastoma (SH-SY5Y) cells damaged by okadaic acid. Additionally, the BBB permeation was predicted using parallel artificial membrane permeability assays.

## Results and discussion

### Neuroprotective effects of the different extracts rich in crocins

**Fig. 1:**
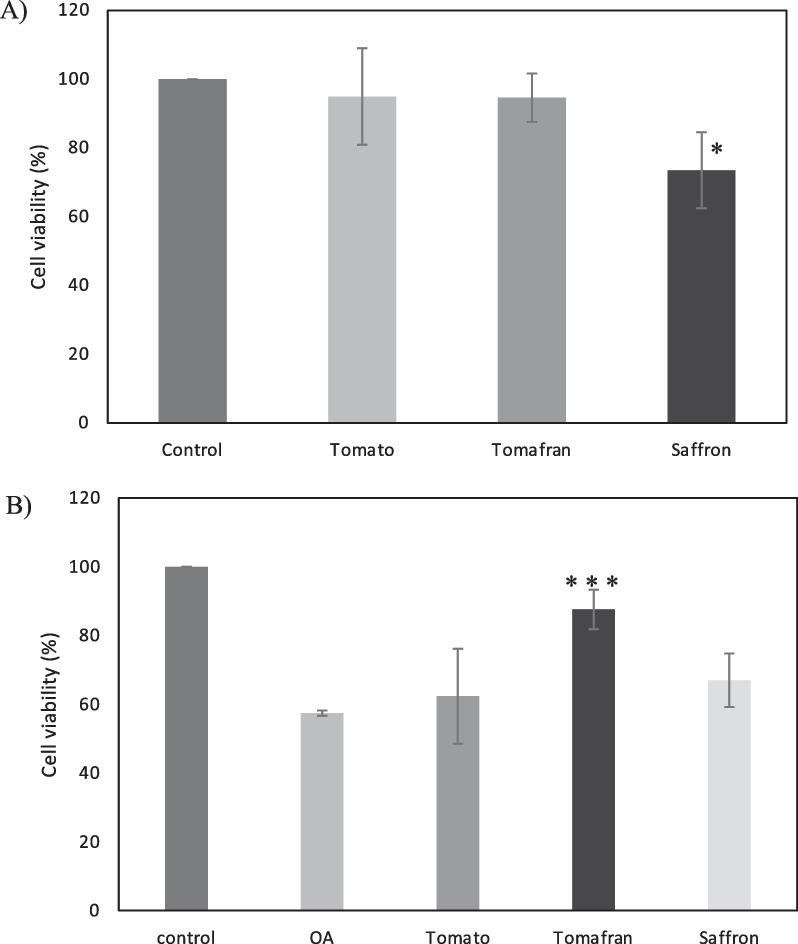
**A** Percentage of cellular viability of SH-SY5Y using tomato, Tomafran and saffron extracts. **B** Percentage of cellular viability of SH-SY5Y after OA treatment, using tomato, Tomafran and saffron extracts. (*P < 0.05, **P < 0.01, ***P < 0.001). (One-way ANOVA with a post hoc Tukey test)

With the aim to evaluate the potential neuroprotective activity, different extracts rich in crocins have been evaluated. Tomato fruits from wild type and bio-engineered tomato were examined for the presence of saffron apocarotenoids (crocetin, crocins, picrocrocin, and safranal) after the seeds had been removed. The tomatoes from both lines were crushed and the serum juice was collected and lyophilized. As a control, a water extract from saffron dried stigma was used. Crocins were quantify by HPLD-DAD, and extracts from saffron and Tomafran were diluted to a concentration of 100 µM of crocins while the tomato extract was adjusted to the corresponding amount of Tomafran by weight. Then, these extracts were studied in a cellular model of early staged of Alzheimer’s disease (AD) characterized by high phosphorylation of microtubule-associated protein tau. It is well known that treatment of neuroblastoma SH-SY5Y cells with okadaic acid (OA), a protein phosphatase inhibitor, produces neurotoxicity by high molecular weight phospho-tau species [18], being the cellular model here used. Firstly, and to check that the extracts were not toxic for the cells, cell viability after extracts treatment was measured using the MTT (3-(4,5-dimethylthiazol-2-yl)-2,5-diphenyl-2H-tetrazolium bromide) method. The results showed that both tomato extracts did not reduce cell viability, when a 100 µM of crocin in Tomafran extract was used, however saffron extract reduced cell viability by 27% (Fig. 1A).

Treatment with 70 µM of OA was used to inhibit the activity of protein phosphatases in undifferentiated neuroblastoma SH-SY5Y cells that express tau endogenously, decreasing the cell viability of about 43%. Only Tomafran extract was able to reverse the cell death significantly, pointing out its neuroprotective effect against this toxin (Fig. 1B), and showing its potential as herbal treatment, food supplement or nutraceutical for AD, being safer than saffron extracts.

### Prediction of CNS penetration of the different extracts rich in crocins

**Fig. 2:**
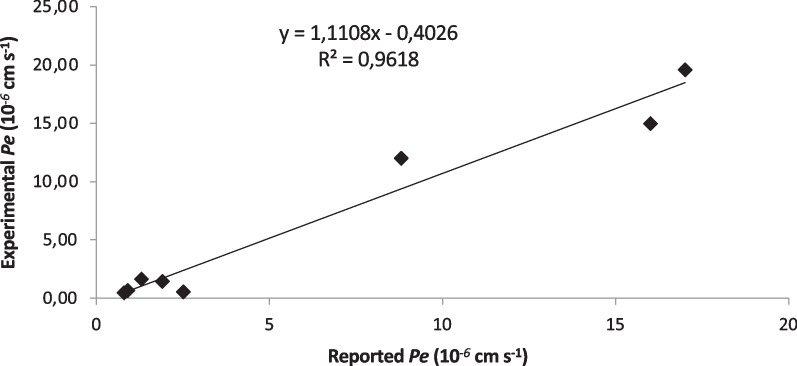
Linear correlation between experimental and reported permeability of commercial drugs using the PAMPA-BBB assay

As previously mentioned in the introduction part, one of the main obstacles for the treatment of the diseases of the central nervous system (CNS) is the drug’s penetration into the brain at therapeutic concentrations. Most CNS drugs cross the BBB by transcellular passive diffusion, due to the tight junction structure and limited transport pathways. Parallel artificial membrane permeability assay (PAMPA) is a high throughput technique developed to predict passive permeability through biological membranes. In order to explore the capacity of tomato, saffron and Tomafran extracts to penetrate into the brain, a PAMPA-BBB method employing a brain lipid porcine membrane was carried out [19]. The in vitro permeabilities (*Pe*) of ten commercial drugs for human therapy through lipid membrane extract together with tomato, saffron and Tomafran extracts were determined and described in Table 1. An assay validation was made comparing the reported permeabilities values of commercial drugs with the experimental data obtained employing this methodology. A good correlation between experimental-described values was obtained *Pe* (exptl) = 1.1108 (bibl)—0.9618 (R^2^ = 0.961) (Fig. 2). From this equation and following the pattern established in the literature for BBB permeation prediction [20], we could classify compounds as CNS + when they present a permeability > 4.04 × 10^–6^ cm s^−1^. Based on these results we can consider that tomato, saffron and Tomafran extracts are not able to cross the BBB by passive permeation (Table 1).

**Table 1:** Permeability (*Pe* 10^–6^ cm s^−1^) in the PAMPA-BBB assay for 10 commercial drugs (used in the experiment validation) and tomato, saffron and Tomafran extracts with their predictive penetration in the CNS

Compound	Bibl.^a^	*Pe* (10^–6^ cm s^−1^)^b^	Prediction
Atenolol	0.8	0.5 ± 0.3	
Caffeine	1.3	1.7 ± 0.4	
Enoxacin	0.9	0.7 ± 0.6	
Hydrocortisone	1.9	1.4 ± 1.1	
Ofloxacin	0.8	0.5 ± 0.3	
Piroxicam	2.5	0.6 ± 0.8	
Promazine	8.8	12.0 ± 0.9	
Testosterone	17	19.6 ± 0.1	
Verapamil	16	15.0 ± 0.2	
Tomato		0.6 ± 0.1	CNS −
Saffron		1.2 ± 0.1	CNS −
Tomafran		0.8 ± 0.2	CNS −

PBS:EtOH (70:30) was used as solvent. ^a^Reference Di et al. ^b^Data are the mean ± SD of 2 independent experiments. CNS−: not able to cross the BBB by passive permeation

However, previous reported results showed that crocins cross the BBB by passive permeation at higher time-points [21]. Furthermore, crocins are digested in the colon to produce deglucosylated trans-crocetin, which is then rapidly and extensively absorbed beyond the intestinal barrier via passive transcellular diffusion [22]. In this sense, we decided to test in the PAMPA-BBB assay, Tomafran and previously digested Tomafran extract for 4, 8, 12, 16, 20 and 24 h. Again, an assay validation was made comparing the reported permeabilities values of commercial drugs with the experimental data obtained employing this methodology. A good correlation between experimental-described values with the corresponding equation was obtained (Table 2).

**Table 2:** Obtained correlation (r^2^) for equations of each time-point experiment

Experiment hours	r^2^
4	0.9876
8	0.9342
12	0.939
16	0.9475
20	0.9571
24	0.9668

As previously described, compounds were classified as CNS +/±/− depending on the obtained equation. The results indicated that Tomafran extract is not able to cross the BBB at any of the tested times. Meanwhile, the previously digested extract of Tomafran was able to penetrate the BBB within 20 h with a *Pe* (10^–6^ cm s^−1^) of 5.20 which may indicate that trans-crocetin might permeates BBB with a slow but constant velocity over a 24 h period in agreement with reported studies (Table 3). Anyhow, better strategies for brain delivery would be desirable.

**Table 3:** Tomafran extract and digested Tomafran with their predictive penetration in the CNS at 4, 8, 12, 16, 20 and 24 h

Sample	Prediction (4 h)	Prediction (8 h)	Prediction (12 h)	Prediction (16 h)	Prediction (20 h)	Prediction (24 h)
Tomafran	CNS −	CNS −	CNS −	CNS −	CNS −	CNS −
*Pe* (10^–6^ cm s^−1^)	1.82 ± 0.19	0.84 ± 0.15	0.45 ± 0.04	0.93 ± 0.63	0.79 ± 0.48	0.34 ± 0.25
Tomafran (Digested)	CNS −	CNS −	CNS −	CNS ±	CNS +	CNS +
*Pe* (10^–6^ cm s^−1^)	0.98 ± 0.77	2.04 ± 0.52	1.19 ± 0.86	5.20 ± 1.09	7.04 ± 0.65	13.20 0.25

### Tomafran extract encapsulation into chitosan nanoparticles and in vitro cellular studies

In order to improve the delivery of Tomafran extracts into the CNS, we decided to encapsulate the extract in chitosan nanoparticles. Polymeric nanocarriers (NCs) based on low MW chitosan are potential candidates for the non-invasive conveyance of therapies via the BBB [23, 24] having many benefits, such as low toxicity, biodegradability, biocompatibility, and mucoadhesivity. Moreover, chitosan has other advantages such as the simplicity of functionalization due to functional groups on its structure, which further enhances the capabilities of chitosan-based NCs. Therefore, to improve the velocity of permeability we decided to encapsule Tomafran extracts (TFE) into chitosan (CH) nanoparticles.

Different nanoparticles formulations were obtained using two CH:TFE ratios formulations 5:1 and 1:1. To determine the correct proportions of raw material and plant extract, photonic spectroscopy to measure of average size, surface Z potential, and polydispersity index (PDI) of each ratio formulations was used. The results presented in Table 4, showed nanoparticles average size ranges from 49 to 106 nm of diameter; showing an increase of the size of the nano vehicle related to the TFE encapsulation. However, nanoparticles (NPs) with the ratio 1:1 and 5:1 shown similar size in agreement with previous works reporting that plant extracts chitosan nanoparticles did not displayed a significative increase of average size due to a higher molecular weight of raw material [25]. Polydispersity (PDI) index was measured to know the homogeneity of nano-formulations, Table 4 shows the decrease of PDI from 0.4 to 0.3 in NPCH (chitosan nanoparticles) and NPCH-TFE (chitosan nanoparticles containing Tomafran extract) formulations respectively. The incorporation of glycosylated apocarotenoids could provide a stabilizing effect due to the alkyl glycosidic structure conferring non-ionic surfactant properties [26].

**Table 4:** Average size, polydispersity (PDI), Z-potential, encapsulation efficiency (EE) and loading capacity (LC) for NPs characterization

Formulation	NPCH-TF formulations				LC (Crocins) µM
	Size (Rh.nm)	PDI	Z potential	EE%	
NPCH	48.6 ± 3.4	0.4 ± 0.2	+ 31.4 ± 1.4	–	–
NPCH-TFE 5:1	104.3 ± 1.6	0.3 ± 0.0	+ 26.5 ± 0.8	38.7	216
NPCH-TFE 1:1	106.3 ± 1.8	0.3 ± 0.0	+ 24.2 ± 0.8	84.4	338

Data are expressed as mean ± s.e.m. from at least three independent experiments

Surface Z potential is a predictive value of stability and quality of nano-formulations, where values near to + 30 mV and − 30 mV indicates a stable colloid suspension [27]. Furthermore, the surface charge is indicative of the electrochemical properties of the nanoparticles, since the type of charge provides different physicochemical and biological properties, besides different approaches to surface chemical modifications of the nanoparticles. The results showed in Table 4 presented a positive surface charge with an interval ranging from + 31.4 to + 24.2 mV in NPCH and NPCH-TFE 1:1 respectively. Nanoparticles showed a characteristic positive surface Z potential related to the use of chitosan raw material and non-ionic surfactants with suitable values close to + 30 mV indicating a well stabilized suspension. The decrease of surface zeta potential has been related to the increase of TFE into chitosan nanoparticles in agreement with previous works where the increase of active molecule in chitosan nanoparticles decrease the surface charge [28].

**Fig. 3:**
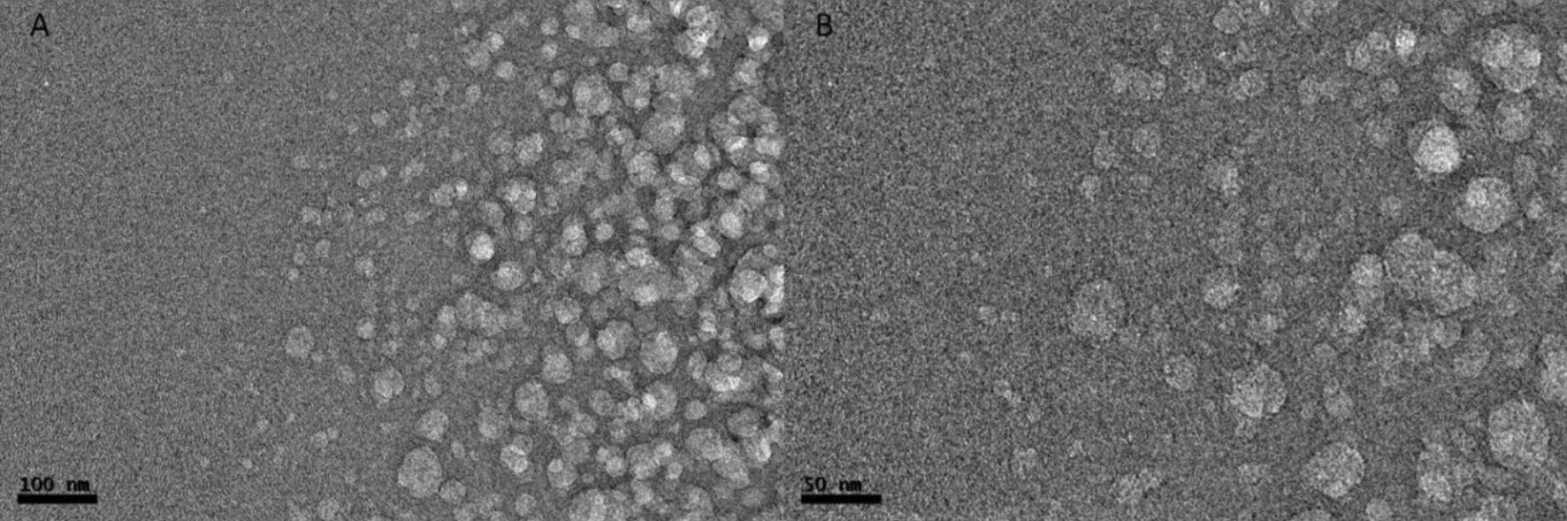
**A** NPCH-TFE image with 100 nm scale bar. **B** NPCH-TFE image with 50 nm scale bar

Finally, transmission electron microscope (TEM) was used to determine the surface and morphological characteristic of chitosan nanoparticles [29]. The images in Fig. 3 corresponding to two different TEM micrograph magnifications showing in both cases spherical shaped nanoparticles with some aggregates. The size of the nanoparticles is distributed between 10 and 30 nm and differs from the size obtained by Dynamic Light Scattering (DLS), confirming the swelling and aggregation effect observed previously in other works with chitosan nanoparticles due to self-aggregation among individual nanoparticles and or/swelling of the chitosan layer surrounding the nanoparticle in aqueous media. Because of these phenomena, the size and PDI measurements in DLS show a higher size than the TEM technique [30].

**Fig. 4:**
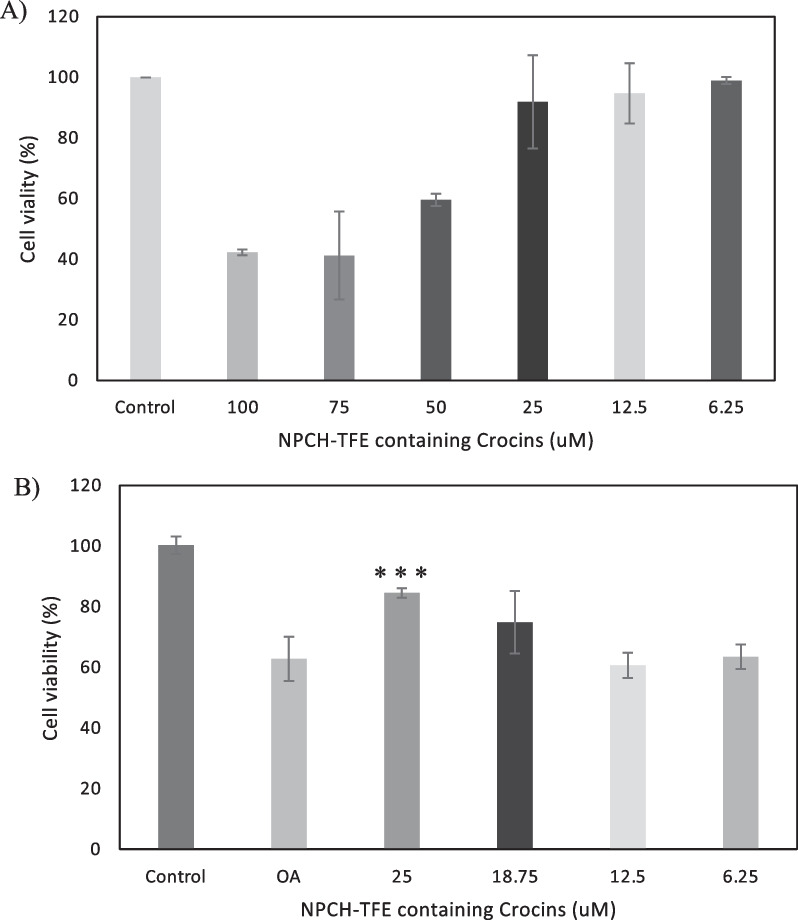
**A** Percentage of cellular viability of SH-SY5Y using NPCH-TFE containing different concentration of crocins. **B** Percentage of cellular viability of SH-SY5Y after OA treatment, using different crocin’s doses of NPCH-TFE. Asterisks denote significant differences between OA group and treated groups (*P < 0.05, **P < 0.01, ***P < 0.001). (One-way ANOVA with a post hoc Tukey test)

Once the NPCH-TFE were obtained and physico-chemically characterized, different concentration of encapsulated Tomafran extracts ranging from 100 to 6.25 μM were assayed to check SH-SY5Y cell viability. The formulations NPCH-TFE 5:1 and NPCH-TFE 1:1 have been assessed using SH-SY5Y cells in the presence of okadaic acid (OA), and the results obtained were comparable among them. Only data from NPCH-TFE 1:1 are presented. As shown in Fig. 4A, NPCH-TFE 1:1 containing concentration from 50 to 100 μM were toxic to SH-SY5Y cell and reduce the cell viability by an average of 50% while NPCH-TFE 1:1 containing lower concentration of crocins were harmless to cells. Therefore, neuroprotection assays against OA at doses ≤ 25 µM crocins were carried out. As shown in Fig. 4B, the neuroprotective activity of NPCH-TFE 1:1 at 25 µM is statistically significant.

Finally, the artificial membrane permeability test (PAMPA) was carried out under the same previously described conditions. The new formulations NPCH-TFE 5:1 and NPCH-TFE 1:1 were able to cross the barrier by passive diffusion with a *Pe* (10^–6^ cm s^−1^) of 14.8 ± 1.8 and 14.4 ± 3.4, respectively. Compared to the TFE solution, passive penetration of NPCH-TFE was significantly improved. Neither TFE membrane retention nor binding to the filter's surface occurred. Thus, the data show that the NPCH-TFE formulations successfully ameliorated passive permeation trough the BBB.

### Characterization of tomato and Tomafran derivate exosomes

**Fig. 5:**
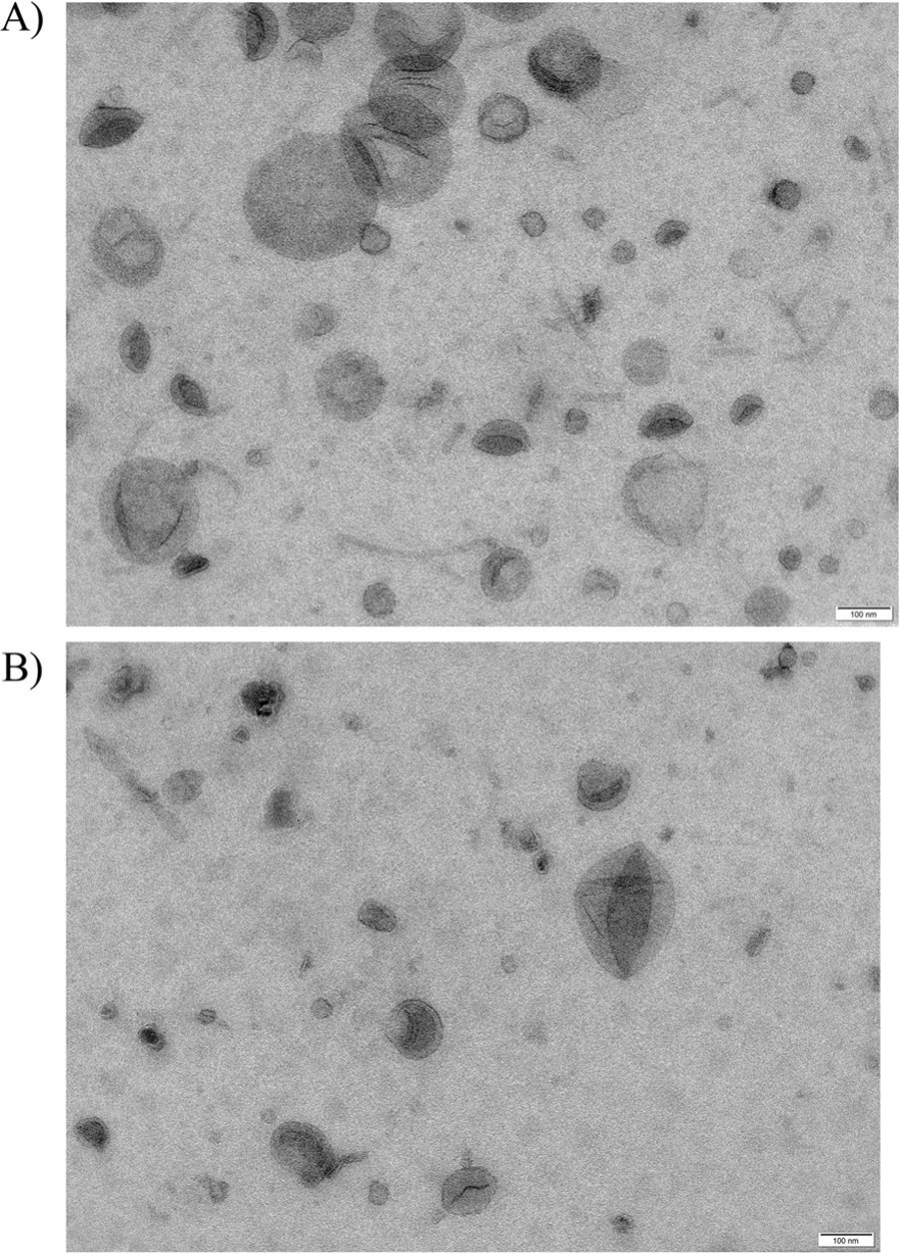
Scanning electron micrographs of fixed and dehydrated exosomes from wild type tomato (**A**) and Tomafran (**B**)

**Fig. 6:**
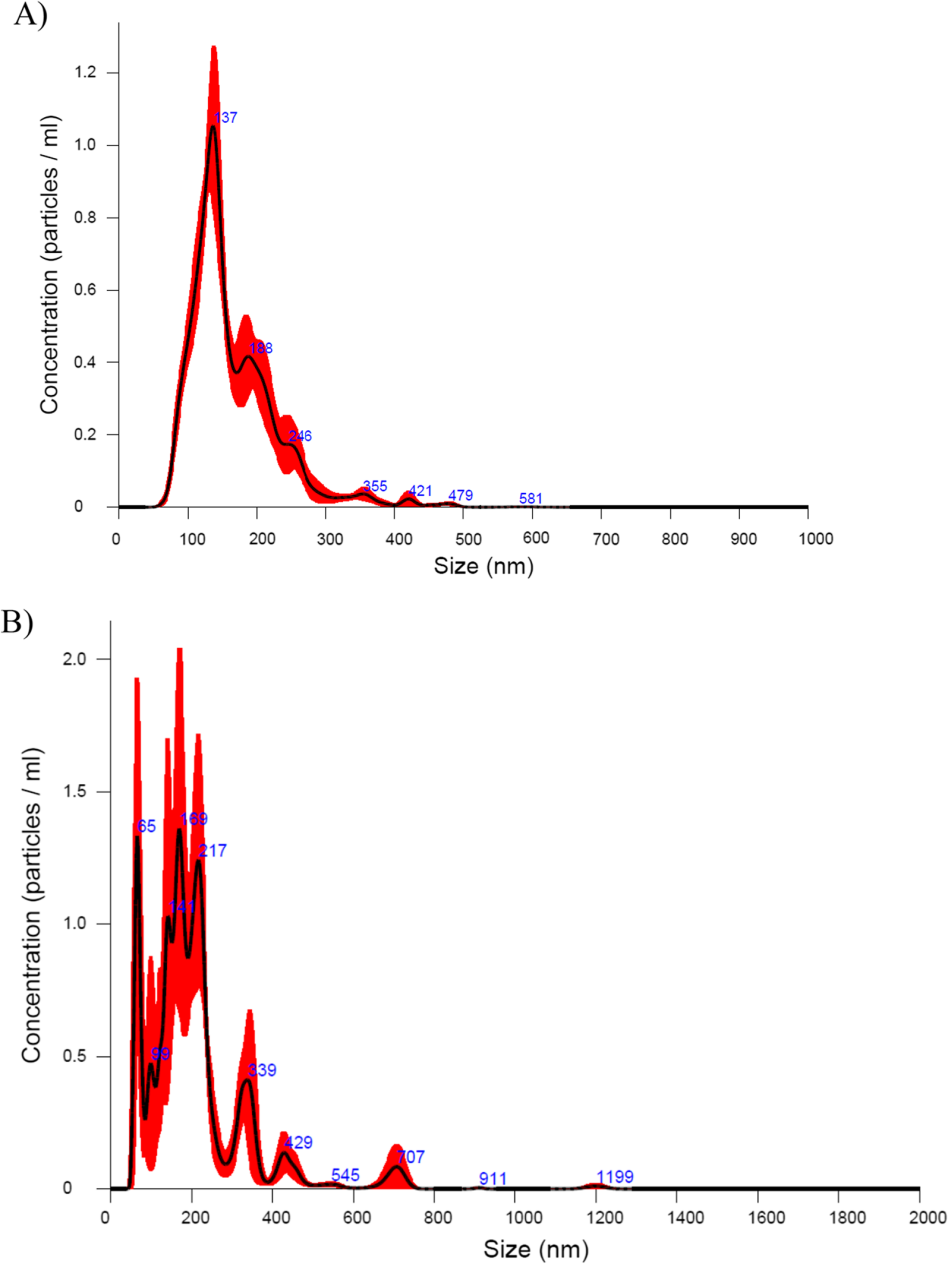
Quantification of tomato money maker (**A**) and Tomafran (**B**) vesicle concentration by nanoparticle tracking analysis (NTA)

In addition to NPCH-TFE, we decide to also study the neuroprotective capacity of tomato and Tomafran exosomes. Exosomes originating from particular plant sources, such as ginseng or green tea, have been the subject of some studies looking into their therapeutic potential in Alzheimer’s disease animal models. These investigations have shown encouraging findings, indicating that plant exosomes may have neuroprotective qualities and may be employed as therapeutics [31]. For all the above reasons, we aimed to purify exosomes from tomato wild type and Tomafran to find out whether they have a potential neuroprotective activity. In this work, we collected and screened two plant-derived exosome from a wild-type tomato and transgenic Tomafran in order to offer a new alternative to treat CNS disorders based on these natural extra vesicles. Before the in vitro cytotoxicity evaluation, we first characterized the different isolation of exosomes from tomato and Tomafran using TEM (Fig. 5) and nanotraking analysis (NTA) (Fig. 6).

To have a closer look at the morphology of these vesicles, TEM analysis confirmed the presence of vesicular structures. Preparations of exosomes with a final protein concentration of 1.9 mg/mL in Tomafran and 3.7 mg/mL in tomato were used. To have a closer look at the morphology of these vesicles, we performed a TEM analysis that confirmed the presence of vesicular structures. Both exosomes preparations have an average size of 140 nm (Fig. 5) with a typical cup-shaped morphology. Most exosomes are spherical, but sample fixation and dehydration result in cup-shaped morphology observed by SEM and TEM [32].

NTA is a widely technique that permits the determination of both the size distribution and relative concentration of microvesicles, including exosomes, in the supernatants of cultured cells and biological fluids [33]. The NTA of purified exosomes are shown in Fig. 6. In both cases, the exosomes displayed a wide size distribution of particles with higher number of vesicles less than 200 nm achieving a maximum concentration of particles with 137 nm and second minor population with 188 nm in the case of wild type tomato exosomes. Meanwhile, the exosomes population in transgenic Tomafran showed three main populations of nanovesicles with 65 nm, 169 nm and 217 nm. The distribution observed in the case of tomato exosomes was similar to the profile obtained recently by Kilasoniya and co-workers where the exosomes obtained from tomato juice showed a main population of PELNs ranging from 140 to 170 nm [34]. Another recent work described a similar pattern in the size distribution of exosomes obtained from tomato confirming the main population with exosomes with 180 nm [35].

**Fig. 7:**
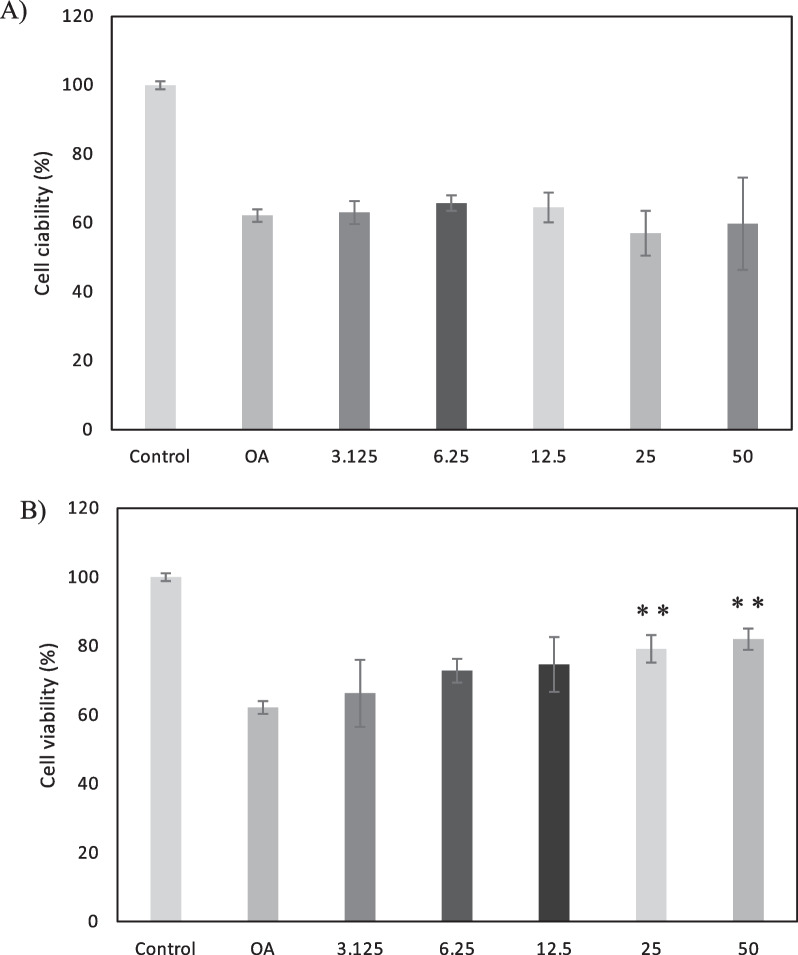
Percentage of cellular viability of SH-SY5Y after OA treatment, using (**A**) exosomes from Tomato wild type (**B**) Tomafran. Asterisks denote significant differences between OA group and treated groups (*P < 0.05, **P < 0.01, ***P < 0.001). (One-way ANOVA with a post hoc Tukey test)

Neuroprotection studies were done with different concentrations of Tomafran exosomes as shown in Fig. 6. Results shown that exosomes from tomato wild type were not able to protect the SH-SY5Y cells from the damage caused by OA while exosomes from Tomafran at doses of 25 and 50 μg/mL protect the cells significatively (Fig. 7). This effect may be due to the presence of crocins which exert its strong antioxidant activity by reducing oxidative stress and inflammation.

The use of plant exosomes in a therapeutic setting, either by leveraging known therapeutic capabilities of plants or by packing plant exosomes with a therapeutic cargo, is a recurring future prospect.

## Conclusions

In this study, we have shown that Tomafran extracts, chitosan nanoparticles of tomato rich in crocins and exosomes from Tomafran are good candidate to act as a neuroprotectants against toxicity induced by okadaic acid trough tau phosphorylation. Our findings support the importance of nanoparticles to delivery drugs since they are able to cross the BBB and put the spot on the exosomes derived from plant, which might be a source of neuroprotective vesicles. Future research will be necessary to support the findings and elucidate the key components and additional mechanisms underlying exosomes and Tomafran neuroprotective benefits. These materials may be used as herbal therapy, food supplement or nutraceutical for delay AD progression and/or its prevention.

## Experimental section

### Plant material

Wild type and transgenic tomato [*Solanum lycopersicum* cv. Moneymaker (MM)] were treated and cultivated as previously described [36]. Tomato fruits were collected at the ripe stage and tissues, once separated from the seeds, were cut into pieces, and immediately frozen in liquid nitrogen until further analyses.

### Tomato extracts preparation

Wild-type (TWTE) and transgenic tomato (Tomafran, TFE) were independently crushed in a blender, the obtained juice was centrifuged at 9000*g* for 15 min. The liquid phase was collected and lyophilized. The lyophilized of wild-type and transgenic extracts were weighted, resuspended in distilled water, and diluted to a concentration of 100 µM of crocins. Crocins were separated and identified as previously described [9].

### Cell culture

Human neuroblastoma (SH-SY5Y) cells were purchased from the European Collection of Cell Cultures (Health Protection Agency, Salisbury, UK), and were grown in Dulbecco’s Modified Eagle Medium containing l-glutamine (2 mM), 1% nonessential amino acids, 10% fetal bovine serum, and 1% penicillin/streptomycin under humidified 5% CO_2_ conditions at 37 °C. Upon achieving semiconfluency, cells were pretreated with saffron, tomato and Tomafran extracts, encapsulated extracts and exosomes isolated from TWTE and Tomafran, and then exposed 1 h later to okadaic acid (70 μM) for 24 h. After the incubation time, cultures were processed for the cell viability assay. Cell viability was determined by the MTT assay, as previously described [37]. Cell survival was normalized to untreated controls and is presented as a percentage.

### Tomafran extract encapsulation in chitosan nanoparticles (NPCH-TF)

Chitosan nanoparticles (NPCH) were formulated via ionic-gelation method described by [38] with some modifications. Briefly, CH solution at 0.2% was prepared dissolving CH flakes in acetic acid at 1% under continuous stirring overnight. Then, the CH solution was sonicated 10 min until completely dissolved. 50 mL of CH solution was mixed at 1000 RPM in a 1% Tween 80 solution and heated at 50 °C. After cool the solution, the CH solution was adjusted to pH 6 with NaOH 1 M. Then, different ratios of Tomafran extract (TFE) (from 20 mg-100 mg) were dissolved in 2 mL of MeOH:H_2_O (1:1) solution and were added to 20 mL of CH solution and the emulsion was left to stir for 20 min. Finally, tripolyphosphate (TPP) aqueous solution at 0.2% was added dropwise at 2 mL/min under continuous stirring to induce the ionic gelation. Afterwards, agitation was carried out at 700 RPM for 40 min. The nanoparticles were collected after centrifugation at 15,000 RPM for 20 min at 4 °C, and, subsequently, washed several times with mQ water.

### NPCH-TFE nanoparticles characterization

The encapsulation loading efficiency was obtained by collecting the supernatant of NPCH-TFE in centrifuge tube, filtering through 0.45 µm Millipore filter this fraction was analyzed using high performance liquid chromatography-diode array detector-high resolution mass spectrometry (HPLC-ESI-HRMS) and HPLC–DAD as previously described [39].

The characterization of nano-formulations [size, zeta potential, and polydispersity index (PDI)] was determined by dynamic light scattering (DLS) using a Zetasizer (3000 HSM Malvern Ltd., Madrid, Spain) with the following specifications: chitosan refractive index (IR) of 1.700, absorption index 0.010, and water solvent RI: 1.33, with a viscosity of 0.8872 cP. Measurements were performed in triplicate.

The morphology of NPCH-TFE was carried out using high-resolution electron microscope images of NPCH-TFE on a Jeol JEM 210 TEM microscope operating at 200 kV and equipped with an Oxford Link EDS detector. The resulting images were analyzed using Digital Micrograph™ software from Gatan.

### CNS penetration: in vitro parallel artificial membrane permeability assay (PAMPA)-blood brain barrier (BBB)

Brain penetration prediction of the several studied extracts was evaluated using a parallel artificial membrane permeability assay (PAMPA) [19]. Ten commercial drugs, phosphate buffer saline solution at pH 7.4 (PBS), DMSO and dodecane were purchased from Sigma, Acros organics, Merck, Aldrich and Fluka. The porcine polar brain lipid (PBL) (catalog no. 141101) was from Avanti Polar Lipids. The donor plate was a 96-well filtrate plate (Multiscreen® IP Sterile Plate PDVF membrane, pore size is 0.45 µM, catalog no. MAIPS4510) and the acceptor plate was an indented 96-well plate (Multiscreen®, catalog no. MAMCS9610) both from Millipore. Filter PDVF membrane units (diameter 30 mm, pore size 0.45 μm) from Symta were used to filter the samples. A 96-well plate UV reader (Thermoscientific, Multiskan spectrum) was used for the UV measurements. Test compounds [(3–5 mg of Caffeine, Enoxacine, Hydrocortisone, Desipramine, Ofloxacine, Piroxicam, Testosterone), (12 mg of Promazine) and 25 mg of Verapamile and Atenolol] were dissolved in DMSO (250 µL). 25 µL of this compound stock solution was taken and 225 µL of DMSO and 4750 µL of PBS pH = 7.4 buffer was added to reach 5% of DMSO concentration in the experiment. These solutions were filtered. The acceptor 96-well microplate was filled with 180 μL of PBS/DMSO (95/5). The donor 96-well plate was coated with 4 µL of porcine brain lipid in dodecane (20 mg mL^−1^) and after 5 min, 180 μL of each compound solution was added. 1–2 mg of every extract to be determined their ability to pass the brain barrier were dissolved in 250 µL of DMSO and 4750 µL of PBS pH = 7.4 buffer, filtered and then added to the donor 96-well plate. Then the donor plate was carefully put on the acceptor plate to form a “sandwich”, which was left undisturbed for 2 h and 30 min at 25 °C. Moreover, selected samples were tested at 4 h, 8 h, 12 h, 16 h, 20 h and 24 h at 25 °C. During this time the compounds diffused from the donor plate through the brain lipid membrane into the acceptor plate. After incubation, the donor plate was removed. UV plate reader determined the concentration of compounds and commercial drugs in the acceptor and the donor wells. Every sample was analyzed at three to five wavelengths, in 3 wells and in two independent runs. Results are given as the mean [standard deviation (SD)] and the average of the two runs is reported. 10 quality control compounds (previously mentioned) of known BBB permeability were included in each experiment to validate the analysis set.

### Isolation and purification of plant exosomes from tomato and Tomafran

Extracellular vesicles were isolated from tomato and Tomafran. Fruits were picked from plants growing in greenhouse. Fresh fruits were carefully washed, peeled, and mixed with an equal mass of PBS 1X, and later they were processed with a potato masher. To remove residual debris, the mixture was centrifuged at 500*g* for 10 min, the obtained supernatant was centrifuged at 2000*g* for 20 min, repeating the same procedure at 5000*g* for 40 min and 10,000*g* for an hour. The final supernatant was ultracentrifuged at 150,000*g* for 2 h. The pellet obtained after the ultracentrifugation was washed once with PBS. The washed pellet was resuspended in PBS. The suspension of exosomes was ultracentrifuged using a sucrose gradient with 60, 45, 30 and 15% (p/v) of concentration, the interface was collected, precipitated by centrifugation, and the pellet was washed three times to remove sucrose. Finally, protein content was determined by PierceTM BCA Protein Assay Kit (Thermo) according to the manufacturer’s protocol. Tomato exosomes were finally stored at − 80 °C until used.

### Nanoparticle tracking analysis (NTA), total protein concentration measurement and TEM imaging of exosomes

NTA was performed using the Malvern NanoSight NS300 system on GDENs fractions and dilute 400–2000 folds with 1× PBS to obtain optimal signal count per frame according manufacturer instruction (30–50 reads/frame). Three 60-s videos were recorded and analyzed by NTA software.

### Extracellular vesicles characterization by transmission electron microscopy (TEM)

Transmission electron microscopy was employed to characterize the morphology of tomato exosomes. All of the TEM measurements have been performed by depositing 20 µL of suspension of vesicles on a 300-mesh copper grid for electron microscopy covered by a thin amorphous carbon film. Samples have been deposited at room temperature. Negative staining was realized by addition of 10 µL of 2% aqueous phosphotungstic acid (PTA) solution (the pH was adjusted to 7.3 using 1-M NaOH). Measurements were carried out by using a FEI TECNAI 12 G2 Twin (FEI Company, Hillsboro, OR, USA), operating at 120 kV and equipped with an electron energy loss filter (Biofilter, Gatan Inc, Pleasanton, CA, USA) and a slow-scan charge-coupled device camera (794 IF, Gatan Inc, Pleasanton, CA, USA).

### Statistics

For the study of the plant material, three to five biological replicates with three technical replicates per biological replicate were analyzed for every experiment. The obtained data were statistically analyzed with one-way analysis of variance and Duncan’s test of significance using the SPSS software. Levels of significance were determined by tukey-test.

## Data Availability

The data supporting our findings are available in the manuscript file or from the corresponding author upon request.
